# A Cellularized Biphasic Implant Based on a Bioactive Silk Fibroin Promotes Integration and Tissue Organization during Osteochondral Defect Repair in a Porcine Model

**DOI:** 10.3390/ijms20205145

**Published:** 2019-10-17

**Authors:** Vanessa Pérez-Silos, Nidia K. Moncada-Saucedo, Víctor Peña-Martínez, Jorge Lara-Arias, Iván A. Marino-Martínez, Alberto Camacho, Víktor J. Romero-Díaz, María Lara Banda, Alejandro García-Ruiz, Adolfo Soto-Dominguez, Humberto Rodriguez-Rocha, Norberto López-Serna, Rocky S. Tuan, Hang Lin, Lizeth Fuentes-Mera

**Affiliations:** 1 Departamento de Bioquímica, Facultad de Medicina, Universidad Autónoma de Nuevo León. Madero y Dr. Aguirre Pequeño S/N, Mitras Centro, Monterrey 64460, Mexico; vanessa_silos@msn.com (V.P.-S.); monkda27@gmail.com (N.K.M.-S.); acm590@hotmail.com (A.C.); alexgruiz@hotmail.com (A.G.-R.); 2 Universidad Autónoma de Nuevo León (UANL), Servicio de Ortopedia y Traumatología, Hospital Universitario “Dr. José E. González”, Monterrey 64460, Mexico; doctorviko@hotmail.com (V.P.-M.); jorgelara77@gmail.com (J.L.-A.); 3 Universidad Autónoma de Nuevo León (UANL), Unidad de Terapias Experimentales, Centro de Investigación y Desarrollo en Ciencias de la Salud, Monterrey 64460, Mexico; amarinomtz@gmail.com; 4 Universidad Autónoma de Nuevo León (UANL), Departamento de Patología, Facultad de Medicina, Monterrey 64460, Mexico; 5 Universidad Autónoma de Nuevo León (UANL), Unidad de Neurometabolismo, Centro de Investigación y Desarrollo en Ciencias de la Salud, Monterrey 64460, Mexico; 6 Universidad Autónoma de Nuevo León (UANL), Departamento de Histología, Facultad de Medicina, UANL, Monterrey 64460, Mexico; vikjavi5@hotmail.com (V.J.R.-D.); ibqasoto@yahoo.com.mx (A.S.-D.); humbertordz54@hotmail.com (H.R.-R.); 7 Universidad Autónoma de Nuevo León, Facultad de Ingeniería Mecánica y Eléctrica, Monterrey 66451, Mexico; marialarabanda@yahoo.com.mx; 8 Universidad Autónoma de Nuevo León (UANL), Departamento de Embriología, Facultad de Medicina, Monterrey 64460, Mexico; norbertolopezserna@yahoo.com.mx; 9 Department of Bioengineering, Swanson School of Engineering, University of Pittsburgh, Pittsburgh, PA 15261, USA; rst13@pitt.edu; 10 Center for Cellular and Molecular Engineering, Department of Orthopaedic Surgery, University of Pittsburgh School of Medicine, Pittsburgh, PA 15219-3143, USA; 11 Department of Orthopaedic Surgery, McGowan Institute for Regenerative Medicine, University of Pittsburgh, School of Medicine, Pittsburgh, PA 15260, USA; hal46@pitt.edu

**Keywords:** biphasic scaffold, extracellular cartilage matrix, silk fibroin, osteochondral tissue engineering

## Abstract

In cartilage tissue engineering, biphasic scaffolds (BSs) have been designed not only to influence the recapitulation of the osteochondral architecture but also to take advantage of the healing ability of bone, promoting the implant’s integration with the surrounding tissue and then bone restoration and cartilage regeneration. This study reports the development and characterization of a BS based on the assembly of a cartilage phase constituted by fibroin biofunctionalyzed with a bovine cartilage matrix, cellularized with differentiated autologous pre-chondrocytes and well attached to a bone phase (decellularized bovine bone) to promote cartilage regeneration in a model of joint damage in pigs. BSs were assembled by fibroin crystallization with methanol, and the mechanical features and histological architectures were evaluated. The scaffolds were cellularized and matured for 12 days, then implanted into an osteochondral defect in a porcine model (*n* = 4). Three treatments were applied per knee: Group I, monophasic cellular scaffold (single chondral phase); group II (BS), cellularized only in the chondral phase; and in order to study the influence of the cellularization of the bone phase, Group III was cellularized in chondral phases and a bone phase, with autologous osteoblasts being included. After 8 weeks of surgery, the integration and regeneration tissues were analyzed via a histology and immunohistochemistry evaluation. The mechanical assessment showed that the acellular BSs reached a Young’s modulus of 805.01 kPa, similar to native cartilage. In vitro biological studies revealed the chondroinductive ability of the BSs, evidenced by an increase in sulfated glycosaminoglycans and type II collagen, both secreted by the chondrocytes cultured on the scaffold during 28 days. No evidence of adverse or inflammatory reactions was observed in the in vivo trial; however, in Group I, the defects were not reconstructed. In Groups II and III, a good integration of the implant with the surrounding tissue was observed. Defects in group II were fulfilled via hyaline cartilage and normal bone. Group III defects showed fibrous repair tissue. In conclusion, our findings demonstrated the efficacy of a biphasic and bioactive scaffold based on silk fibroin and cellularized only in the chondral phase, which entwined chondroinductive features and a biomechanical capability with an appropriate integration with the surrounding tissue, representing a promising alternative for osteochondral tissue-engineering applications.

## 1. Introduction

Articular hyaline cartilage has alow repair ability; for this reason, when a lesion advances, it extends to the underlying subchondral bone, generating an osteochondral defect (OC), which leads to a complex therapy [1].

The articular cartilage has an architecture defined not only by the type of proteins of the extracellular matrix ECM but by its arrangement, and it varies from the surface to the subchondral bone; this is known as an osteochondral unit. The histological structure of the osteochondral unit is divided into 4 regions defined according to the content of the proteoglycans, as well as the density of the chondrocytes as follows: (1) Superficial or tangential; fine region aligned parallel to the articular surface, composed mainly of type II collagen fibers; (2) Intermediate or transitional; it has a low density of chondrocytes with a spherical morphology, rich in proteoglycans and thick collagen II fibers; (3) Deep or radial, which is formed by chondrocytes organized with a columnar orientation; and finally, (4) a calcified layer of hypertrophic chondrocytes capable of attaching the cartilage to the bone by anchoring the collagen fibers with the subchondral bone [2,3].

When a defect with a critical size occurs in the osteochondral unit, degenerative changes are induced both in the cartilage and in the bone surrounding the lesion; these changes generate a mechanical destabilization that translates into a decrease in the load properties [4]. Consequently, due to the complex architecture of the chondral tissue, the repair of osteochondral defects requires an approach based on tissue engineering.

Strategies based on tissue engineering offer a promising therapy for the restoration of articular cartilage, and thus, different designs have been proposed. Its principle involves the combination of stem or differentiated cells, scaffolding materials and bioactive factors that facilitate the repair of cartilage. This seems to be convenient for a direct use of the cells on the site of the lesion, in relation to the problem of cell delivery. However, a challenge on which efforts should be invested consists in improving the integration of the implant with the surrounding tissue, as well as the generation of a new tissue that mimics the osteochondral hierarchical structure.

In recent years, the use of biphasic scaffolds has been intensified because they positively influence the hierarchical organization of osteochondral tissue; additionally, there is increasing evidence that these designs favor the integration of the implant with the surrounding tissue [5,6].

Concerning biomaterials of a protein nature, silkworm fibroin is an ideal candidate. Fibroin is a natural biopolymer that is highly biocompatible and biodegradable, which allows it to be used not only in a wide variety of biomedical devices but in an interesting way in the development of new regeneration technologies [7].

In its semicrystalline structure, fibroin has a highly ordered phase of β-antiparallel sheets that give it resistance and toughness; on the other hand, interleaved separators of less-ordered β sheets are placed, contributing to the flexibility and elasticity of the material [8].

Among the biological properties of fibroin, we highlight the ability to sustain the proliferation and differentiation of various cell types, including the chondrogenic lineage; This makes it an attractive biomaterial in regenerative cartilage medicine [9,10].

The use of silk fibroin has been expanded due to its versatility in generating different types of scaffolding, ranging from films to scaffolds. The selection of manufacturing methods and even the use of different solvents in the solubilization of fibroin influence induction capacities toward specific cell lineages such as chondrogenic [11].

For the functionality of a biomaterial within a complex structural context such as the osteochondral one, it is necessary to adapt the properties of the biomaterials to the needs of the chondral or bone tissue. To overcome this issue, biofunctionalization is presented as a valuable procedure.

In this sense, biofunctionalization with an ECM derived from several tissues and decellularized organs has become more attractive and is used for the regeneration of tissues, such as skeletal muscle [12], blood vessels [13], nerves [14], and cartilage [15,16]. The ECM provides informative signals and a unique composition that mimics the natural tissue environment and leads to proper tissue regeneration. In articular cartilage, chondrocytes are surrounded by a highly hydrated ECM consisting of type II collagen (Col II), proteoglycans, and several other proteins, which play a crucial role in chondrogenesis [17,18].

In this study, the design of a biphasic scaffold based on the assembly of a cartilage phase consisting of silk fibroin, biofunctionalized with a bovine cartilage matrix, cellularized with differentiated pre-chondrocytes from adipose tissue stem cells (autologous) and well-attached to the bone phase (decellularized bovine bone) is described and evaluated in terms of mechanical and chondroinductive features. Furthermore, we evaluated its ability to generate well-organized hyaline cartilage and subchondral bone, 4 months after its implantation in an osteochondral lesion in a porcine model.

## 2. Results

### 2.1. Decellularization of Bone and Cartilage; and Assembly of the Biphasic Implant

**Figure 1:**
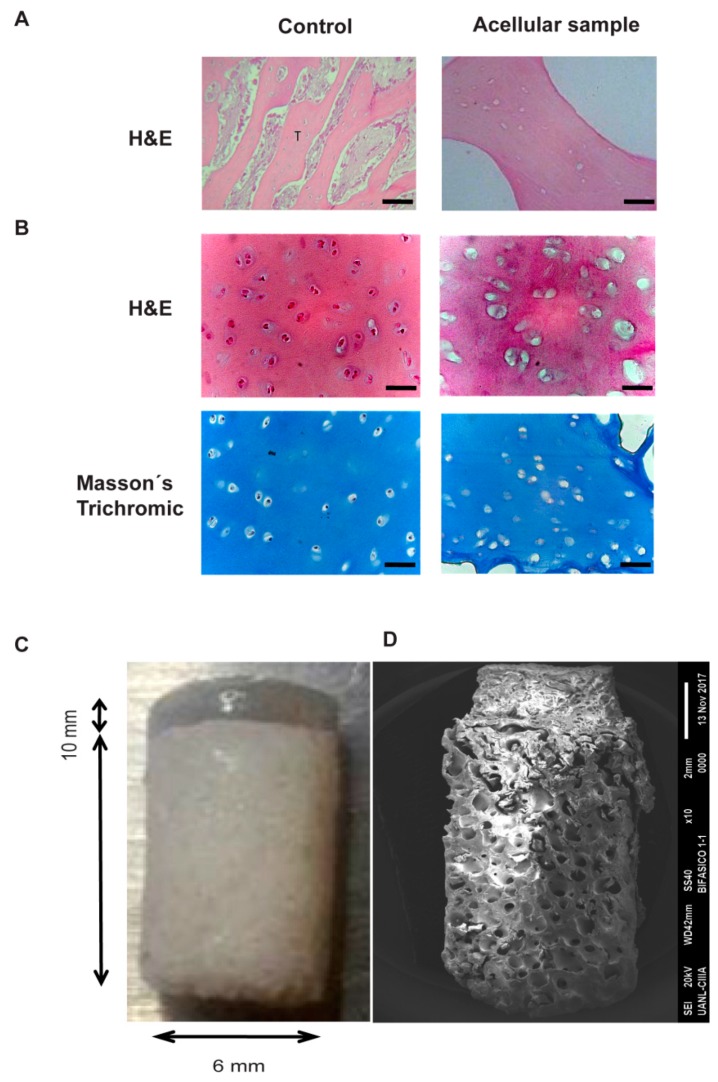
Decellularization of bone and cartilage, and biphasic scaffolding based on fibroin. The decellularization was assessed via an analysis of the H&E stained sections of (**A**) bone and (**B**) cartilage. Furthermore, in (**B**), the integrity of the cartilage matrix was appreciated by Massón’s trichromic stain. Scale bar = 50 µm. (**C**) The gross morphology of the biphasic acellular implant based on fibroin. The photographic image shows the dimension of the scaffolds. (**D**) The scanning electron microscope image shows the porous structure of the cartilage/bone scaffold.

Bovine bone and cartilage were decellularized in order to avoid any immunological response using physicochemical methods, as described in the material and methods section, and then the process efficiency was evaluated by histological staining. As shown in Figure 1A, the complete decellularization and preservation of the extracellular matrix integrity was observed for the bone, so that 8 mm × 10 mm bone chips were obtained. Regarding the cartilage, staining with H&E (Figure 1B) evidenced that the acellular tissue did not contain any cellular component compared to the control, as demonstrated by the presence of empty lacunae in the tissue section. The procedure did not alter the integrity of the ECM, as shown in Figure 1B, according to the presence of the collagen with a regular and dense arrangement stained with Masson’s Trichrome.

After the decellularization of the bone and cartilage and the lyophilization of the cartilage ECM, biphasic scaffolds formed by a chondral phase (CP) and a bone phase (BP) were assembled. The photographs show the dimensions of the constructed implant, as well as the proportion between the chondral and bone phases (Figure 1C). For the fibroin-based chondral phase, NaCl with a particle size of 74–177 µm was used for the generation of the controlled porosity and interconnectivity and was attached to the bone chips via the crystallization of the fibroin with methanol. In the SEM image, it is further observed that the scaffolds had a uniform macro-porous morphology with an evident trademark between the chondral and bone phases (Figure 1C).

### 2.2. Influence of Particle Size of the BCM on the Mechanical Properties of the Chondral Phase

**Figure 2:**
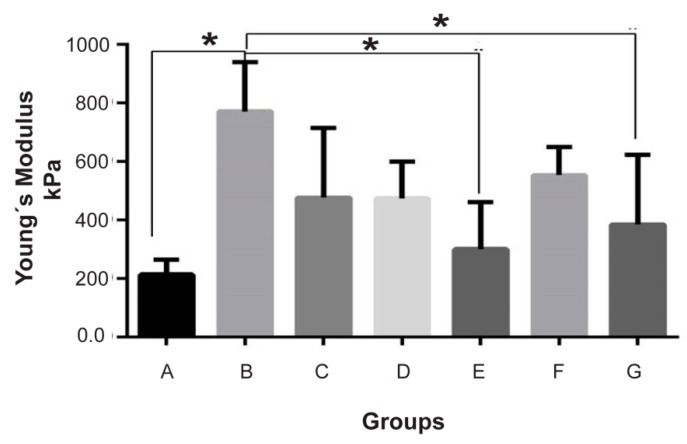
The effect of BCM particle size on the mechanical properties of silk fibroin scaffolding. The determination of the Young’s modulus of fibroin and NaCl (as porogen) hydrogels, and biofunctionalized with the BCM of two different particle sizes; the tested proportions were 1:1, 2:1 and 3:1. The error bars represent the standard deviation; * *p* ≤ 0.05 based on the ANOVA analysis.

The biofunctionalization of fibroin was based on the enrichment of the biomaterial with a bovine cartilage matrix (BCM). To evaluate whether the particular size of the BCM and the proportion of fibroin/NaCl:BCM exerted an influence on the mechanical properties of the chondral phase; two particular sizes (10–20 µm and 20–100 µm) in the proportions 1:1, 2:1, and 3:1,were evaluated by Young’s modulus. The Young’s modulus of the scaffolds is presented in Table 1. Based on our data, all the prepared scaffolds had the ability to keep their original shape after the mechanical load was removed. The Young’s modulus of the scaffolds made without BCM, and also having a small particle size (10–20 µm), was lower than the reported modulus of the native articular cartilage (500–800 kPa) [19,20]. Interestingly, implants constructed with a larger particle size (20–100 µm) showed improved mechanics, even similar to the native articular cartilage. We emphasize that, although no statistically significant differences were observed between the different proportions within this group, the scaffold constructed with an equimolar amount between the porogen (NaCl) and the BCM had a suitable mechanical performance. (Figure 2).

Since the mechanical properties of the biomaterial are a key aspect in the design and selection of the final implant, subsequent experiments only involved the groups constructed with a particle size of 20–100 µm, which means Groups B, C and D.

### 2.3. Pore-Size Analysis in the Cartilage Phase of the Biphasic Implant

**Figure 3:**
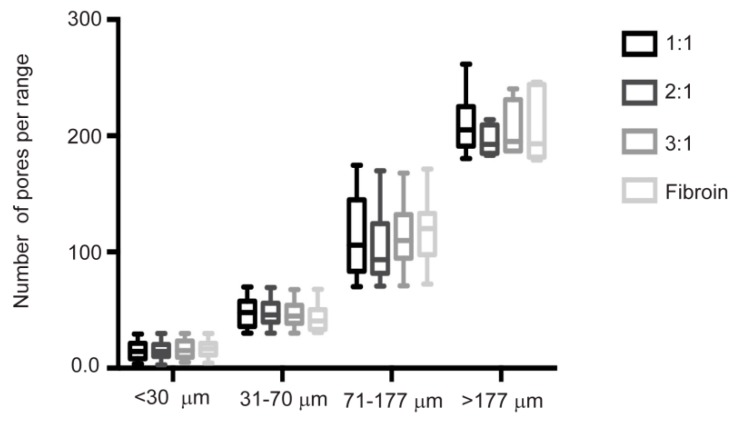
Box-whisker plots showing the pore size distribution for the cartilage phase of the biphasic implant. The graph displays the porosymmetric analysis of the scaffold, expressed as the relative abundance of each pore size in a representative volume. The data are reported as the mean ± SD.

Scaffolds constructed with a BCM with a larger particle size showed elastic properties similar to native cartilage. Since the porosity also influences the mechanical properties of the implants, the range in the pore size was analyzed between Groups B, C and D (proportions 1:1, 2:1 and 3:1, respectively). The analysis of the pore size distribution was performed from the SEM images by measuring the pore diameter size with the Image J program. The analysis results were divided into the following sizes: micropores, <30 and between 31 and 70 µm, related to the promotion of cell adhesion; 71–177 µm, a range that favors anoxia, therefore promoting chondrogenesis; >177 µm, a size related to improving cell proliferation and migration. In the graph, the boxes represent the number of pores that were counted, where the horizontal line represents the average in each size range and the whiskers represent the maximum and minimum size of each pore found per group. A homogeneous distribution between the groups with pore sizes <70 was evidenced; in contrast, the distribution in the range of 71–177 µm, which is related to an efficient chondrogenesis, was favored in the 1:1 ratio. Regarding the pores >177 µm, we observed that the 1:1 ratio, which contained a greater amount of BCM, had a greater number of pores in this range compared to the other proportions; however, the average between them was similar. As expected, in the silk fibroin group (with the porogenic particle), the presence of large pores was prevailing (Figure 3).

### 2.4. In Vitro Response of Chondrocytes in Biofunctionalized Biphasic Scaffolds

It is well documented that the biofunctionalization of biomaterials improves their chondroinductive properties. To evaluate the effect of BCM on the in vitro differentiation of chondrocytes, pre-chondrocytes differentiated from isolated porcine ADSCs were cultured in the biphasic scaffolds with different proportions of fibroin/NaCl:BCM for 28 days, and the content of GAGs, the expression and characteristics of the formed extracellular matrix, and the expression of the ColII and ColI proteins were all evaluated.

**Figure 4:**
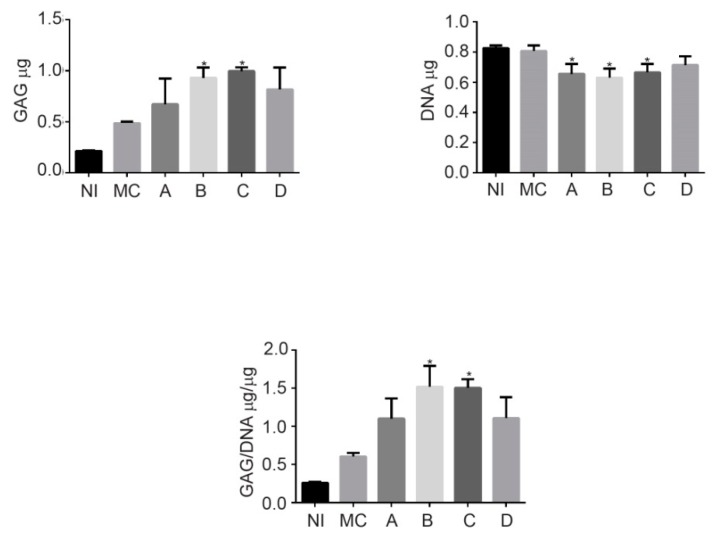
The matrix production by GAGs (µg), normalized to the total DNA content (µg). The data from three independent experiments were evaluated, and the mean ± standard deviation is indicated. * *p* < 0.05 vs. Group A (no biofunctionalized). NI = Non-induced control, MC = Monolayer culture.

The secretion of GAGs clearly increased in the groups cultured in a 3D system (Groups A–D) compared with the secretion of the chondrocytes in a monolayer culture (MC), as shown in the normalized graph against the DNA content (*p* < 0.05); however, no significant differences were observed in the GAG content among the fibroin groups that were biofunctionalized with BCM (Groups B–D) (Figure 4). These results suggest that at 28 days, the increase in the amount of BCM does not induce a higher production of GAGs.

**Figure 5:**
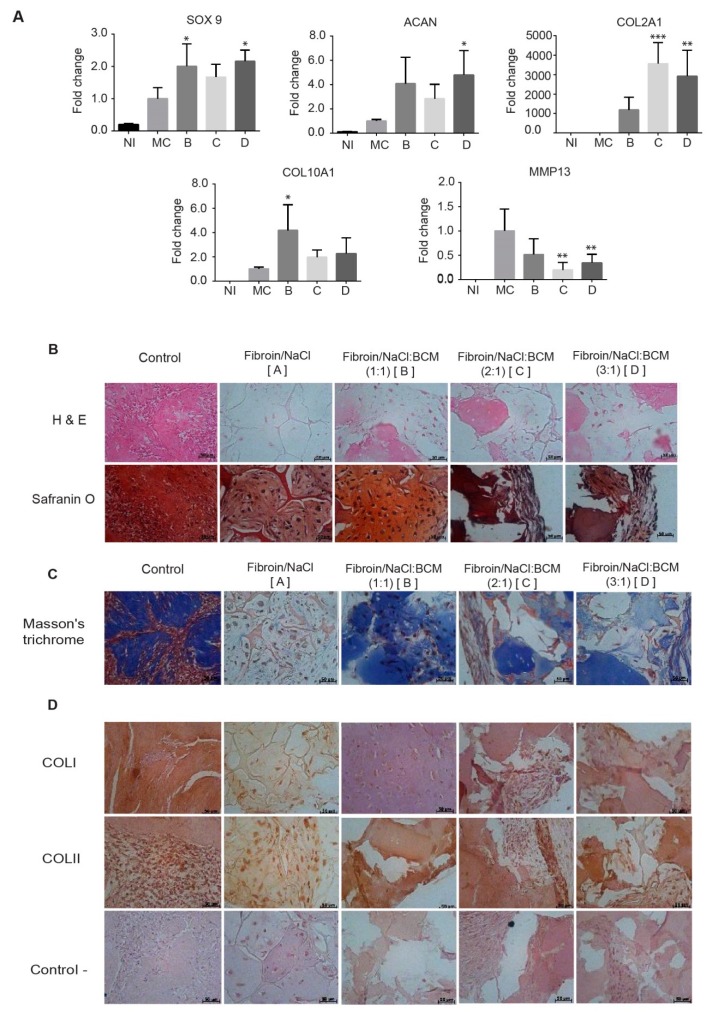
In vitro response of chondrocytes in biofunctionalized biphasic scaffolds. (**A**) Relative gene expression of *SOX9, ACAN COL2A1, COLXA1* and *MMP13*. The data are reported as the mean ± standard deviation (*n* = 3). * *p* < 0.05, ** *p* < 0.01, *** *p* < 0.001 versus MC. NI = Non-induced control, MC = Monolayer culture. Histological evaluation of Groups A to D after 28 days of culture. (**B**) Hematoxylin and eosin (H&E), and safranin O staining showing that the group consisting of fibroin/NaCl:BCM in a ratio of 1:1 showed a structural organization of the neoformed tissue similar to the native chondral tissue. (**C**) Masson’s trichrome staining showed, in the same Group B, a strong positive staining of hyaline cartilage with a good column alignment of chondrocytes, which was similar to the morphology of native cartilage. (**D**) Representative images for type II and type I collagen immunohistochemistry. All the groups showed weak positive staining for type I collagen, as well as a strong signal for type II collagen. Negative controls were treated with PBS without primary antibodies. Scale bar = 50 μm.

To better characterize the influence of BCM on the composition of the extracellular matrix, as well as on the presence of chondrocyte differentiation regulatory genes, the expressions of *SOX-9, ACAN, COL2A1, COLXA1* and *MMP13* in increasing the matrix proportions were analyzed by qRT-PCR. Our analysis showed that the transcription factor *SOX9*, which is the chondrogenic lineage marker, is expressed primarily when cells are grown in a three-dimensional system (Groups B, C and D) instead of a monolayer culture, showing no statistically significant differences when the amount of BCM is increased. We emphasize that, since the measure in the expression is at 28 days, the data indicate that the chondrogenic phenotype remains stable and that the process of dedifferentiation is not observed. Regarding the expression of extracellular matrix protein genes, the aggrecan mRNA expression is not influenced by the proportion of BCM. In contrast, at the transcriptional level, the expression of *COL2A1* shows an inverse relation to the amount of matrix contained in the implant, evidencing an over-expression in the Groups 2:1 and 3:1 compared to 1:1 (Figure 5A). The hypertrophy genes *COLXA1* and *MMP13* were analyzed, showing that at 28 days the monolayer-grown chondrocytes reached a terminal differentiation before those cultivated in the implant; nonetheless, among the different proportions of BCM that were analyzed, no statistically significant differences were found in the expression of these genes (Figure 5A).

To evaluate the significance of the BCM on the structural organization of the neoformed tissue, stains with H&E, Safranin O and Masson’s Trichrome, as well as immunostains for collagen I and II, were performed. The biofunctionalized groups (B, C and D groups) evidence, via H&E, a dense production of neoformed matrix; furthermore, as expected, a low cellularity of typically rounded chondrocytes was also observed. In contrast, in the group without BCM (group A), the neoformed matrix was scarce and dispersed (Figure 5B). The structural organization of sulfated proteoglycans (in red) was evaluated by Safranin O staining. We emphasize that, in Groups A and B, the presence of chondrocytes surrounded by sulfated proteoglycans (red) is observed within a well-organized matrix, while, in contrast, C and D reflect a poorly structured matrix. This observation is relevant since proteoglycans support the stabilization and organization of collagen fibers (Figure 5B).

Following that line of thought, Masson’s trichrome staining was performed to evaluate the presence and distribution of collagen fibers. Interestingly, astounding differences between Group B compared with C and D were detected. In Group B, the presence of well-organized and abundant collagen fibers in the neoformed matrix was confirmed (Figure 5C).

To define the nature of the collagen fibers observed by Masson’s trichrome stain, the immunodetection of type I and II collagen was performed. All the functionalized groups showed an adequate content of Col II when compared to the control of native tissue. Furthermore, when analyzing the content of Col I, Group B evidenced a lower content compared to Groups A, C, D and even the native control, and in consequencea Col II:Col I ratio suitable for the hyaline cartilage (Figure 5D).

Thus, the results suggested that the biphasic implant with a 1:1 ratio of fibroin/NaCl:BCM is able to promote, in cultivated chondrocytes, the generation of a well-organized neoformed matrix rich in sulfated proteoglycans and collagen fibers, with an suitable ratio of Col II:Col I, exhibiting a hyaline-like neoformed tissue.

### 2.5. Implantation of the Constructs

According to the results described above, the structural design of the biphasic scaffold was defined. To further characterize the biphasic implant biofunctionalized with BCM for cartilage tissue engineering and to examine the reparative potential for clinical applications, we used a porcine in vivo model to evaluate the integration of the biphasic implant with the surrounding tissue and the repair process in osteochondral lesions.

The proposed design involves the cellularization of the chondral phase with pre-chondrocytes. In order to evaluate the dependence of the cellularization of the bone phase as a means of making the integration of the implant more efficient, both versions were implanted, without cells and cellularized with osteoblasts differentiated from porcine ADSCs.

**Figure 6:**
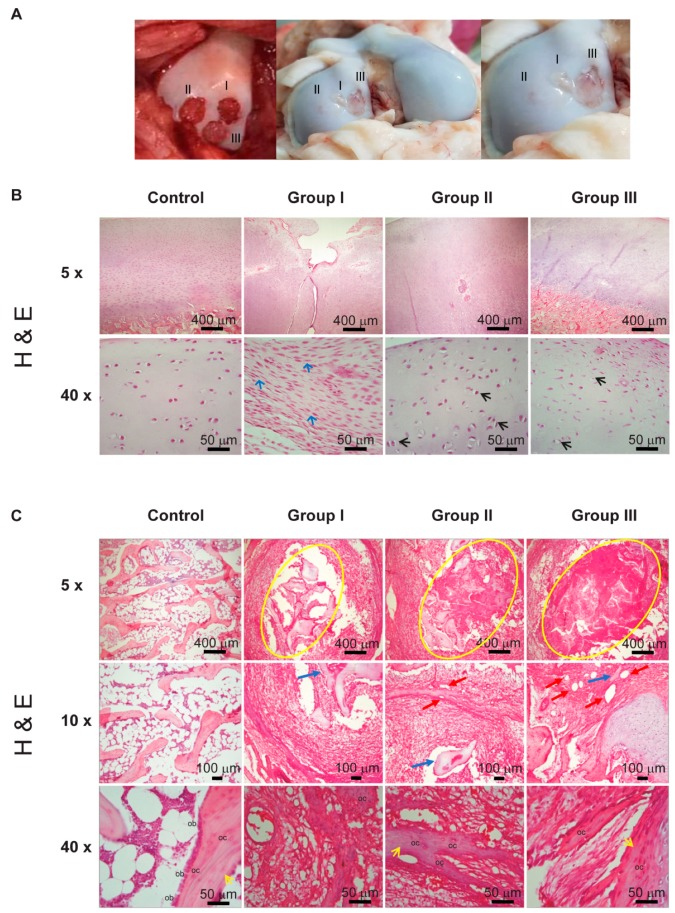
Cartilage gross appearance and H&E staining at 8 weeks post-transplantation. (**A**) Representative images of the gross appearances at 8 weeks after operation. Numbers I, II and III indicate the location of the implants: monophasic, biphasic and cellularized only in the chondral phase, and biphasic and cellularized in both phases, respectively. (**B**) Representative histological images of the repaired chondral area analyzed by H&E at 8 weeks after implantation. In the panoramic image (5×), the degree of filling of the lesion is appreciated, while the 40× magnification evidenced a hierarchical organization similar to the control, where in Groups II and III the presence of lacunae with characteristic spherical chondrocytes (black arrows) can be observed. In contrast, in Group I there is abundant filling tissue, predominantly of cells with a fusiform morphology similar to that of fibroblast cells (blue arrows). (**C**) The area enclosed within the yellow line represents the bone repair area. Groups I, II and III show an adequate integration with adjacent tissue; we noted the presence of osteoblasts (ob) as well as osteocytes (oc) embedded in the bone matrix, with the formation of blood vessels (red arrows) as well as fibroin residues (blue arrows). The groups treated with biphasic scaffolds showed a better cellular and tissue organization compared to the control, highlighting in Group II the presence of trabeculae (yellow arrow).

The implantation surgery was conducted under general anesthesia. Three osteochondral defects were created in the femoral condyles, with an outer diameter of 3 mm by a 9 mm depth. These engrafted sites were labeled as follows: Group I, cellularized monophasic cartilage implant; Group II, a biphasic implant with the chondral phase cellularized; and Group III, a biphasic implant cellularized in both chondral and bone phases (Figure 6A). The day after the surgery, the motion of the knee was not restricted, and full weight-bearing on the surgical joints was allowed. One week after the surgery, no evidence of defects in the march was observed.

### 2.6. Macroscopic Evaluation of Cartilage Repair at 4 Months Post-Engraftment

After 4 months of recovery, the pigs were sacrificed via an overdose of barbiturates. Later, the condyles were recovered. The macroscopic photographs were taken, and the pieces were fixed to proceed with the microscopic analysis. Through the macroscopic examination, it can be seen that in Group II the total thickness defects were repaired, showing a glossy white tissue surface, which was comparable to the appearance of normal articular cartilage (Figure 6A). In contrast, the defects in Groups I and III remained largely unfilled (Figure 6A).

### 2.7. Morphological Analysis

For the microscopic analysis, H&E staining was performed for the observation of the cell morphology and the organization of the connective tissue; likewise, the integration of the scaffolds and the tissue formed de novo with the adjacent tissue. In Figure 6B, the chondral phase in the native cartilage and different treatments are shown. In the cartilage control, round shaped chondrocytes in their lacunae observed, either isolated or in isogenic groups; among these, the capsular or pericellular matrix was identified, followed by a territorial matrix with basophilic staining from the sulfated compounds, and, finally, the interterritorial matrix with acidophilic staining resulting from a large amount of collagen fibers. These findings were compared with the study groups. Group I, in which the lesions were treated with monophasic scaffolds, showed little filling of the lesion and cells with a fusiform morphology similar to that of fibroblast cells (blue arrow), in addition to showing an extracellular matrix with a disorganized appearance.

In contrast, in Groups II and III, a cell morphology similar to that of the control group was observed, i.e., lacunae with characteristic spherical chondrocytes (green arrows), separated by an extracellular matrix.

Interestingly, Group II showed a hierarchical organization of chondrocytes and collagen fibers similar to that of native cartilage.

Underlying the chondral phase, the bone phase was observed through the presence of trabecular or spongy bone tissue (Figure 6C), composed of osseous trabeculae with osteocytes included in the matrix. In the control subchondral native bone, the trabeculae surrounded by lines of cubic cells [(ob) Figure 6B 40×] was observed, as well as osteocytes [(oc) Figure 6B 40×] trapped in the matrix that they secreted.

Regarding the integration of the implant, Figure 6C shows the area of the lesion that had been repaired over 8 weeks; Groups I, II and III show a good integration between the de novo developed tissues and the adjacent tissue. In the repair area shown in panoramic view (Figure 6C Magnification 5×), the group that was treated with the monophasic scaffold again shows a scarce filling, where the bone matrix shows a disorganized neoformed matrix. Notably, in the groups treated with the biphasic scaffolds (Groups II and III), the area of the lesion is practically filled, and there is an evident formation of trabeculae with osteocytes in the lacunae (oc), especially in Group II (yellow arrow), which indicates a better regeneration of the tissue. All groups show remnants of the fibroin material (blue arrows). We highlight the key presence of blood vessels (red arrows), which demonstrates the neovascularization of the de novo tissue.

To evaluate the composition and organization of the neoformed connective tissue in both chondral and bone phases, Masson’s trichrome stains and the periodic acid-Schiff (PAS) method were performed, which stained the collagen fibers in blue and the complex polysaccharides in magenta pink, respectively.

**Figure 7:**
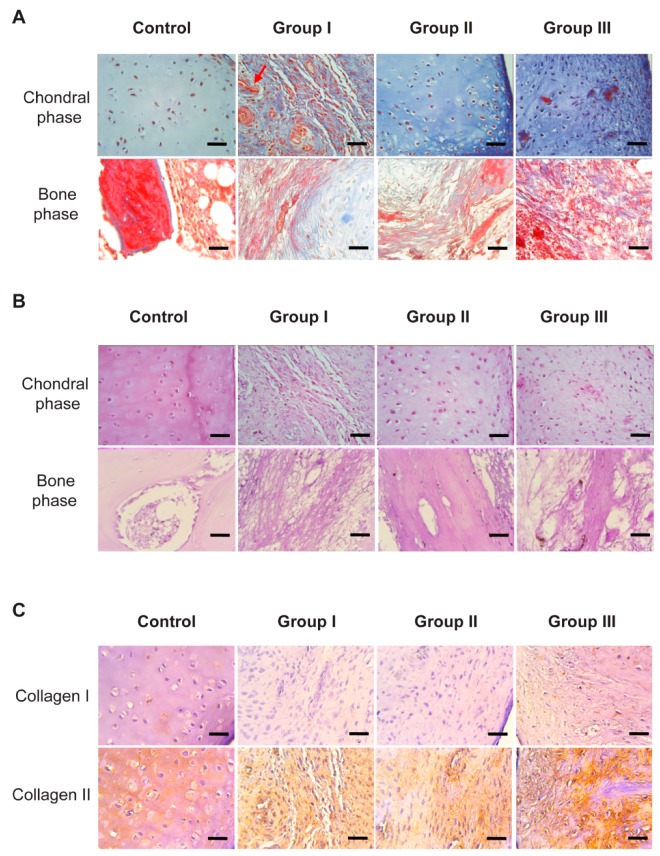
Analysis of repaired tissue at 8 weeks post-transplantation. (**A**) Masson’s trichrome staining; the scaffold of Group II shows a greater organization of collagen fibers. In the chondral phase, the control group is observed with pale staining in the territorial and interterritorial matrices between the chondrocytes. Groups I, II and III have collagen bundles and cells between them, and a greater organization is observed in group II. The presence of blood vessels is indicated by red arrows. In the bone phase, a better organization of the trabeculae in Group II is observed. Scale bar = 50 μm. (**B**) PAS staining; The Group II scaffold induces a greater secretion of complex polysaccharides at the implant site. All the groups show a positive reaction to the histochemistry of PAS; however, it is weaker in the chondral phase of Group I. There is a greater organization of bone tissue in Group II, unlike Groups I and III. Scale bar = 50 μm.(**C**) IHC with anti-collagen I and II; the scaffolding of Group II induces a greater organization of collagen II at the implant site. Chondral phase: a higher content of collagen II than type I was observed in all the experimental groups, similar to the control group. Group II shows a better organization of the fibers in the extracellular matrix. Scale bar = 50 μm.

Through Masson’s trichrome staining, the cartilage control showed a pale blue coloration in the territorial and interterritorial matrices separating the isolated chondrocytes or in the isogenic groups. Interestingly, the chondral phase of Groups II and III showed intense staining of the fibers; in addition, Group II showed an optimal organization of the fibers, mimicking the hierarchical organization of the native cartilage (control), that is, a scarce superficial (tangential) zone followed by the media (transitional) zone. One should note the presence of blood vessels in the chondral phase of Group III, which is not favorable in this tissue. In Group I, bundles of disorganized fibers were observed between the spindle-shaped cells (Figure 7A).

On the other hand, in the bone phase, the presence of trabeculae with collagen fibers, surrounded by endosteum, was confirmed. In the experimental Groups I, II and III, bundles of collagen fibers were observed in the trabeculae, in addition to round cells between them (Figure 7A).

Figure 7B shows positive staining for PAS in the chondral phase in pink; this positivity showed that all the scaffolds of the experimental groups allowed the secretion of proteoglycans, important elements of the fundamental substance of the cartilage. In the control group, positivity was observed in the matrices surrounding the chondrocytes. Similarly, all the experimental groups showed positivity; however, Group I showed a lower positive reaction, as well as showing once again the disorganization of the extracellular matrix. In contrast, Group II showed characteristic spherical chondrocytes, separated by an extracellular matrix with an adequate organization of proteoglycans (Figure 7B).

In the qualitative analysis of the bone phase, an apparent similarity was observed in the PAS histochemistry, positive in all the groups. However, Group II evidenced the clear formation of trabeculae with osteocytes, in comparison with the other experimental Groups I and III, which showed a disorganized morphology of the positive zones (Figure 7B).

Finally, an immunolabelling was performed for the identification of type I and II collagen fibers in the chondral phase (Figure 7C).

It is important to note that a higher content of collagen II than collagen I was observed in the chondral phase at the implant site in all the experimental groups, and this was similar to the control group (Figure 7C).

## 3. Discussion

The aim of this study was to examine the effect of a new biphasic scaffold on the integration with the host and on the tissue organization during osteochondral defect repair.

The biphasic scaffold, based on biofunctionalized fibroin with BCM attached to a bone phase and cellularized only in the chondral phase, showed not only an appropriate integration with the host tissue but also the ideal replacement of the damaged area, with cartilage that maintained the hierarchical structure of the chondral tissue.

Hydrogels, based on various polymeric biomaterials such as PLGA [Poly (lactide-co-glycolide)] and PEG [Poly (ethylene glycol)] or natural materials such as silk fibroin (both capable of generating a three-dimensional network structure), offer multiple advantages in terms of cartilage tissue engineering; these include: (1) a simple and highly reproducible manufacturing methodology; (2) the ability to integrate different types of cells; (3) an adequate biodegradability and biocompatibility to allow cartilage regeneration; and (4) the modulation of mechanical properties [21,22,23].

In this sense, the resistance and tenacity of the fibroinmimic the characteristics of the cartilage, which is not the case for the elastic modulus that, as shown in Figure 2, is naturally inferior to that required by the cartilage to carry out its function.

The biphasic design described here presents a cartilage phase based on fibroin manufactured by crystallization with methanol. On the other hand, the porosity was generated by the leaching method with NaCl as porogen with a particle size between 77 and 177 micrometers, in order to obtain a uniform porous and interconnected structure with pore sizes ranging between 400 and 500 micrometers [24,25,26]. Although, via this method, we obtained a chondral scaffold with an interconnected pore ultrastructure (Figure 1 and Figure 3) that favors adhesion and cell-cell contact, the resulting mechanical properties were still insufficient for the solicitation of the chondral tissue.

To overcome mechanical tissue demands, a strategy used by our group [16] and others has been to incorporate decellularized ECM processed in the form of micronized particles.

Even though the effect of the particle size on the properties of proliferation and differentiation toward adipocytes [27], osteoblasts [28] and chondrocytes [29] has been studied, its impact on mechanical properties and especially on elasticity has been rarely addressed.

In this work, the biofunctionalization of silk fibroin was not only used to recreate the microenvironment of the hyaline cartilage [30] but also to modulate its mechanical properties. Regarding the mechanical features; as shown in Figure 2, the addition of BCM clearly increased Young’s modulus by 2- to 4-fold, and the magnitude of the increase depended on the size of the matrix particle, contributing to a better elastic module than the one that was assembled with a BCM between 2 and 100 µm.

To our knowledge, this is the first research that analyzes the ECM in two different size ranges to evaluate its influence on mechanical properties.

Kun et al. reported that 6% of porcine cartilage matrix nanofibers prepared via the freeze-dried method, with a particle size ranging from 50 to 500 nm, have an elastic modulus of 40.208 ± 5.097 kPa [31]; this is insufficient to cover the requirements of the chondral tissue.

Unlike Kun et al. we compared particle sizes above the range of nanoparticles; and interestingly we observed that implants constructed with a larger particle size (20–100 µm) showed improved mechanics that were even similar to native articular cartilage (Figure 2).

In this research, in addition to studying the effect of the particle size of BCM on Young’s modulus, the effect of three different proportions with respect to the fibroin hydrogel system obtained by the NaCl leaching method was also analyzed.

Rowland et al. studied the effect of different concentrations (between 7% and 11%) of a porcine cartilage matrix, with a particle size no greater than 97 μm, on the mechanical properties of a reticulated collagen system. They observed that only a concentration of 11% is able to resist the deformation of the scaffold (better compression module); however, this affected the porosity and therefore the cellular infiltration [32]. These data confirm that an increase in the concentration of the cartilage matrix does not necessarily lead to a scaffold with good mechanical and biological properties.

In our system, in contrast to the data reported by Rowland, different proportions of the matrix did not exert a statistically significant effect on the mechanical properties of the scaffold (Figure 2).

These discrepancies may be due to differences in the nature of the scaffolding. The manufacturing leaching process provides a controlled porosity for the resulting scaffolding.

Although there were no differences in the Young elastic modulus with respect to the BCM proportions that were analyzed, we certainly do observe a considerable influence of the microenvironment created in different proportions on the behavior of the chondrocytes cultivated in the scaffolds.

It has been shown that the mechanical features of articular cartilage are closely related to two of the main components of the hyaline cartilage matrix: sulfated proteoglycans and collagens [33].

According to Muiznieks et.al., type II, IX and XI collagens are responsible for the shape, tensile stiffness and strength of cartilage tissue [34], while proteoglycans are responsible for their compressive strength [35].

The data presented in Figure 2 and Figure 5 are consistent with this assertion, where the highest content of proteoglycans in Group II conferred a greater elastic modulus. Via safranin O, the presence of chondrocytes surrounded by sulfated proteoglycans was demonstrated within a properly organized matrix. Moreover, Masson’s trichrome stain confirmed the presence of abundant well-organized collagen fibers in the newly formed matrix, where a type II collagen expression predominated, generating a Col II:Col I ratio typical for hyaline cartilage (Figure 5D).

We postulate that this increase of the matrix synthesis in the chondral phase was a combined effect of a homogeneous and interconnected porosity, and the presence of the BCM rich in chondro-inductive signals in a proportion (1:1 ratio of fibroin/NaCl:BCM) that allowed for themaintenance of the scaffolding microstructure.

Although the in vitro models offer valuable information about the behavior of an implant under a controlled environment, they do not give evidence of two aspects that determine the clinical application of an implant: the integration with the surrounding tissue and the organization of the tissue in the area of repair of an osteochondral defect. To date, the important challenge of integrating and maintaining the hierarchical structure of the osteochondral tissue has been poorly addressed [36,37].

In order to generate a scaffold to guide the synthesis and hierarchical organization of the newly formed ECM in the context of osteochondral damage, a biphasic scaffold was designed by tissue engineering and was evaluated in a porcine osteochondral damage model.

In the strategy described here, the pre-chondrocytes (Groups II and III) and the osteoblasts (Group III) were included in scaffolds of a defined size, shape and architecture. The biological similarity of the constructions with the native articular cartilage was demonstrated in Figure 1C. The procedure involved a phase of in vitro maturation of the cellularized implant. This step allowed the embedded cells to begin the synthesis of the ECM under a controlled system, as the presence of abundant type II collagen and GAG demonstrated (Figure 4; this is key, since it has been reported that this profile correlates with the mechanical quality of engineering hyaline cartilage [38]. Moreover, it also encourages the platform on which the host cells are established to preserve the hierarchical organization in the neoformed ECM.

8 weeks after placing the implants, our histological data (Figure 6) showed that only the cellularized biphasic implant in the chondral phase (Group II) had succeeded in establishing the architecture of the osteochondral tissue (Figure 6B and Figure 7A), constituted by an ECM moderately rich in GAGs (Figure 7B) and presenting, toward the surface, a smooth texture (Figure 6A).

The tissue formation of hyaline cartilage was evident in Group II, as evidenced by the presence of lacunae containing cells embedded within the basophilic substance that was rich in GAG and type II collagen. Regarding Groups I and III, fibro-cartilage repair tissue was observed, as indicated by the presence of type I collagen, in addition to the poor secretion of proteoglycans compared to native articular cartilage.

It has already been described that a lesion on the articular surface generally causes the proliferation of chondrocytes in the proximal zone and increases the synthesis of matrix macromolecules. However, this anabolic response of the chondrocytes terminates a short time after the injury, which leads to an inadequate filling of the defect in the tissue [39]. The influence of the biphasic implant, but not the monophasic one, could have supported the compensatory mechanisms of the chondrocytes under a scaffolding system that served as a guide for the hierarchical structure, leading to the production and proper structuring of a matrix rich in proteoglycans and collagens in repaired chondral tissue.

Regarding the bone phase, it has been reported that the created microenvironment is capable of activating the surrounding stem cells into responding, thereby promoting osteogenesis [40,41].

According to the immunohistochemical observation, both in Group II (non-cellularized in the bone phase) and in Group III (cellularized in vitro with osteoblasts), the expression of type I collagen was evidenced, which is an indicator of a good recovery of the osteoblastic tissue, and which also coincides with the expression of type II collagen in the chondral phase, suggesting an adequate repair of the osteochondral tissue as a whole [42].

Consequently, what is the influence of the cellularization of this bone phase in terms of osteochondral tissue repair?

The different patterns of vascularization within the framework of the osteochondral tissue are a major issue. The bone is a highly vascularized tissue, while the cartilage is avascular. The avascularity of the cartilage and also its resistance to the invasion of the vascular networks are important for its function [43]; otherwise, the cartilage can result in ossification of the deep and intermediate zone and consequently exacerbate the joint damage.

As indicated by the H&E staining, in the bone phase of Group II a greater number of nascent blood vessels were observed in comparison with Group III, which suggests that a non-cellularized scaffolding system allows the establishment of host cells for the promotion of an adequate ECM, as well as for the modulation of the vascularization degree. We also noted that the ECM of the cartilage, in this Group II, showed almost no angiogenesis, which benefited the repair of cartilage.

Poor integration has discarded many cartilage repair approaches, and this problem derives from the intrinsic metabolism of cartilage [44].

The ability of the implants to integrate with the host tissue was histologically evaluated. In the case of Group II, the ECM that was produced was integrated with the surrounding tissue, which resulted in a significantly better macroscopic and microscopic healing of the cartilage defects when compared to Groups I and III. No signs of cleft, delamination and fissures were observed at the boundary between the host tissue and the repair tissue (Figure 5).

We postulate that the integration observed in Group II is an additive effect resulting from a scaffolding system that serves as a guide for maintaining the hierarchical structure of osteochondral tissue, as a bone phase capable of promoting cell migration, as the generation of a newly formed tissue that entwines with the collagen fibers in the recipient site and as a vascularization profile that maintains the healthy state of the bone without invading the chondral tissue.

In conclusion, the biphasic implant, based on fibroin and biofunctionalized with BCM in a 1:1 ratio with respect to fibroin/NaCl, develops an ECM rich in sulfated proteoglycans and collagen fibers with an appropriate ratio of Col II:Col I, and with mechanical characteristics similar to native cartilage. The implant, when it is only cellularized in the cartilage phase, is able to repair a full-thickness osteochondral lesion, showing a bright white tissue surface after a period of 4 months. Repair tissue evidenced a hierarchical organization of chondrocytes and collagen fibers similar to that of native cartilage. Taking together, these data show that we can establish that the design described here represents a promising alternative for osteochondral tissue-engineering applications.

## 4. Materials and Methods

### 4.1. Preparation of Natural Bovine Cartilage Matrix (BCM)

Bovine knees explants (thickness = 2 mm) were obtained from six animals (aged <3 years.) in an aseptic environment. The joints were acquired from an abattoir with TIF certification within 24 h of sacrifice. The cartilage was collected from the area of the condyles, patella, and trochlea with a scalpel No. 24 (SensiMedical). The physicochemical decellularization consisted of five cycles of thermal shock in liquid nitrogen for 5 min followed by a wash in PBS for 10 min. Later, the matrix was crushed with a blender. Once crushed, the matrix was washed for 24 h in hypotonic buffer (10 mM TRIS-HCL, 2 mM EDTA, pH 8) supplemented with 100 mM KCl and 5 mM MgCl_2_. Next, hypotonic buffer supplemented with 100 mM KCl, MgCl_2_ and 0.5% SDS was added for 18 h. Finally, the matrix was washed with a hypotonic buffer with 0.5% SDS for 36 h. Following sterilePBS rinsing, the samples were immediately frozen at −80 °C. Before implantation, the samples were lyophilized for 24 h for the complete removal of interstitial fluid, followed by fine grinding. For the pulverization, a pulverizer mill K10 (Micron, Shanghai, China) and a Freezer/mill 6870 (SPEX(R) samplePrep, Metuchen, NJ, USA) were used, generating a particle size of 10–20 µm and 20–100 µm, respectively.

### 4.2. Preparation of Natural Bovine Bone Chips (BBC)

Subchondral bone was collected from bovine knees with trephine 6 mm in diameter. The decellularization consisted of 5 washing cycles in 0.1% SDS in PBS (Sigma-Aldrich, St. Louis, MO, USA) at 100 °C followed by incubation with 30% H_2_O_2_ (Sigma-Aldrich, St. Louis, MO, USA) for 5 h. Subsequently, the bone chips were washed with deionized water in order to remove the remains of SDS and H_2_O_2_. Finally, the decellularized bones were allowed to dry at room temperature and stored until use. Their decellularization by H&E was evaluated.

### 4.3. Biphasic Implant Assembly

For the chondral phase, the silk fibroin was dissolved in Hexafluoro-isopropanol (HFIP) (Sigma-Aldrich, St. Louis, MO, USA) to obtain an 8% solution. The chondral phase was manufactured with fibroin and NaCl (particle size from 74 to 177 µm) in a 1:1 ratio with or without BCM (pulverized with the K10 mill or the Freezer/thousand 6870) at different proportions (NaCl:BCM; 1:1, 2:1 and 3:1). The biphasic scaffold was prepared as follows. The NaCl with or without BCM was placed in cylindrical Teflon molds of 2 cm × 6 mm, and 140 μL of silk fibroin (SF) was added by scaffolding. Subsequently, the BBC (pre-soaked with 90% methanol) was placed into the mold, and 90% methanol (Sigma-Aldrich, St. Louis, MO, USA) was added to induce the crystallization of the SF. The scaffolds in the mold were allowed to stand for 24 h at room temperature. To remove the salt, the scaffolds were washed in ultrapure water for 3 days. Finally, the scaffolds were frozen at −80 °C and lyophilized for 24 h. The sterilization was carried out with ethylene oxide. Seven groups of implants were obtained; Groups B, C and D were manufactured with the pulverized matrix in the Freezer/mill 6870, while Groups E, F, G and H were manufactured with the BCM pulverized with the K10 mill. Group A: SF, Group B: SF/NaCl:BCM 1:1, Group C: SF/NaCl:BCM2:1, Group D: SF/NaCl:BCM3:1, Group E: SF/NaCl:BCM 1:1, Group F: SF/NaCl:BCM 2:1, and Group G: SF/NaCl:BCM 3:1.

### 4.4. Scanning Electron Microscopy

The samples were fixed in 4% glutaraldehyde, and then soaked in 0.1M sodium cacodylate for 10 min and washed three times. The samples were transferred into 1% osmium tetraoxide for 2 h for post-fixation and washed with 0.1M sodium cacodylate. The fixed samples were dehydrated in a graded series of acetone, placed into the critical point dryer (CPD Baltec-030) for 30 min, mounted onto a stub sputtered with gold coating in a Sputter Coater Polaron E-5100 scanning electron microscopy SEM Coating Unit and viewed under a JEOL JSM 6400 scanning electron microscope (JEOL Corporation Ltd., Tokyo, Japan).

### 4.5. Mechanical Testing

An unconfined compression assay of acellular scaffolds (*n* = 6 per group) was performed on circular discs of 3 mm diameter. The mechanical testing of the scaffolds was carried out with a mechanical tester (Bose Electroforce model 3230 SERIES II). The dimension of the scaffolds was measured with a calibrator, and then they were placed between the compressive motor and load cell and subjected to a 10% compression (0.2 mm) at a speed of 0.01 mm/s. The force on the displacement was graphed. Young’s modulus was calculated from the slope of the line. All testing groups in this study consisted of 6 individual samples, and the statistical significance between the groups was determined by a one-way ANOVA followed by the Tukey-Kramer HSD test. Samples with a *p* < 0.05 were determined to be statistically significant.

### 4.6. Porcine Adipose-Derived Stem Cell Isolation and Cell Cultivation

Porcine ADSCs were isolated from adipose tissues obtained from the subcutaneous fat from the caudal area of euthanized adult Yorkshire pigs of 70 kg (*n* = 4). The adipose tissue sample (0.5 g) was minced into small pieces and digested in 0.1% collagenase I (Gibco-Invitrogen, Carlsbad, CA, USA) at 37 °C for 2 h. The collagenase was inactivated by the addition of supplemented DMEM (Dulbecco’s modified Eagle’s medium containing 10% fetal bovine serum (FBS)) (HyClone, Logan, UT, USA), L-glutamine 10 mM and, penicillin (100 U/mL), streptomycin (100 μg/mL) and amphotericin B (0.25 mg/mL) (all from CORNING Cellgro, Manassas, VA, USA). Cells were cultured in a 25 cm^2^ flask at 37 °C and 5% CO_2_. After a week, the non-adherent cells were removed, and the adherent cells were further cultured in supplemented DMEM. The medium was changed every 2–3 days until the monolayer of adherent cells reached 80% confluence.

### 4.7. Cell Differentiation Assays

To confirm the multipotential differentiation ability of cultured porcine ADSCs, cells were cultivated with the different induction media for 14d. For the adipogenic differentiation, ADSCs were grown to 80% confluence in a 4-well chamber (Thermo Fischer Scientific Nunc, Langenselbold, Germany) in the DMEM supplemented with 10% FBS, 1 mM dexamethasone, 1 mg/mL insulin, 0.5 mM 3-isobutyl-1-methylxanthine, and 100 mM indomethacin. After induction, the cultures were fixed in 10% paraformaldehyde and stained with Oil-red-O solution to detect lipid droplets. For the chondrogenic differentiation, the ADSCs were plated at 2 × 10^3^ cells/cm^2^ in a chondrogenic medium containing DMEM (GIBCO, Scotland, UK) supplemented with 0.010 mg/mL of ITS [insulin transferring selenium] (GIBCO, Scotland, UK), 100 nM of dexamethasone (Invitrogen, Carlsbad, CA, USA), 0.001 mg/mL of ascorbic acid and 10 ng/mL of TGFβ1. The cells were fixed with 3% paraformaldehyde and stained with 1% Alcian blue (Sigma-Aldrich, St. Louis, MO, USA) in 0.1 N HCl for 30 min. A blue coloration is indicative of the synthesis of proteoglycans by chondrocytes. To induce osteogenic differentiation, cells were plated at 2 × 10^3^ cells/cm^2^ in an osteogenic medium that contained the DMEM (GIBCO, Scotland, UK) supplemented with 10% FBS, 10 mM β-glycerophosphate, 0.25 mM ascorbic acid, and 10^−8^ M dexamethasone (Sigma-Aldrich, St. Louis, MO, USA). The phenotype was confirmed using Alizarin red staining to evaluate calcium-rich deposits. Control cultures of ADSCs without differentiation medium were also maintained simultaneously (non-induced group).

### 4.8. Cellularization of Scaffolds and In Vitro Maturation

The ADSCs in passage 3 were cultured in a monolayer with supplemented DMEM at 37 °C and 5% CO_2_ until reaching 90% confluence; subsequently, the cells were pre-differentiated in the chondrogenic medium during 5 d and then trypsinized with 0.25% trypsin (GIBCO, Scotland, UK). Scaffolds of 3 mm in diameter by 9 mm deep were cellularized after hydration with supplemented DMEM for 2 h. The chondral phase was cellularized by injection with 0.5 × 10^6^ pre-chondrocytes in 10 μL of supplemented DMEM. The scaffolds were placed in low adhesion 48-well plates and incubated at 37 °C and 5% CO_2_ for 2 h to allow the cells to adhere to the scaffold. This procedure was followed for the scaffolds of Groups I, II and III. For Group III, after chondral cellularization, the scaffolds were cellularized with osteoblasts. Previously, the ADSCs were differentiated in the osteogenic medium during 5 d and then trypsinized with 0.25% trypsin (GIBCO, Scotland, UK). The bone phase was cellularized by injection with 2.25 × 10^6^ osteoblasts in 20 μL of osteogenic medium and incubated for 2 more hours to allow the cells to adhere to the scaffold. The ones from Groups I and II were grown in low adhesion 48-well plates in a chondrogenic medium, while those in Group III were incubated in a two-chamber bioreactor [42], which allowed the chondral phase to be cultivated in a chondrogenic medium and the bone phase in an osteogenic medium, preventing the media from mixing. All groups were cultured for 12 days for maturation at 37 °C in 5% CO_2_ before being implanted.

### 4.9. Quantification of GAGs, Normalized with the DNA Content

The content of sulfated GAGs was quantified using a Blyscan™ sGAG Assay Kit (B1000; Biocolor, Carrickfergus, UK). The samples were minced into small pieces and digested with the papain extraction reagent (Sigma-Aldrich, St. Louis, MO, USA), L-Cysteina-HCl (Sigma-Aldrich, St. Louis, MO, USA), sodium acetate (Sigma-Aldrich, St. Louis, MO, USA) and EDTA (Sigma-Aldrich, St. Louis, MO, USA) at 65 °C for 18 h. For the DNA quantification, the Quantico^TM^ PicoGreen^TM^ dsDNA Assay Kit (Thermo Fischer Scientific Nunc, Langenselbold, Germany) was used. The fluorescence was read on a plate reader (Biotek) at an excitation length of 485 nm and an emission of 520 nm.

### 4.10. RNA Extraction and Quantitative PCR

RNA (100 ng) was isolated from the chondrocyte/scaffold composites after 28 days in culture using the TRIzol reagent (Life Technologies, Carlsbad, CA, USA). 1 μg cDNA was amplified in a PCR mixture including SYBR Green Realtime PCR Master Mix-Plus (Applied Biosystems, Foster City, CA, USA) and gene-specific primers (Table 2), in accordance with the manufacturer’s information. The reaction was performed using a Step One Real-Time PCR System (Applied Biosystems, Foster City, CA, USA). GAPDH was used as an endogenous control for the study. The relative gene expression profiles were normalized to a GAPDH expression and analyzed using the 2^−ΔΔCT^ approach (*n* = 3).

### 4.11. Histology and Immunohistochemistry (IHC)

After decalcification with 10% formic acid (Sigma-Aldrich, St. Louis, MO, USA), the samples were processed via a conventional histological technique until their inclusion in paraffin blocks. Subsequently, histological sections of 4 μm were stained with hematoxylin and eosin (H&E) for the analysis of the general histology, with safranin O to detect the presence of sulphated proteoglycans, and with Masson’s trichrome for the staining of connective and muscular tissue components, as well as with the acid histochemical technique Periodic Schiff (PAS). This technique selectively identifies the complex polysaccharides (glycosaminoglycans, proteoglycans, and adhesion glycoproteins) present in the extracellular matrix. These samples were analyzed by light microscopy. An immunohistochemical analysis was performed on sections of 4 μm for the specific identification of type I and II collagen fibers in the chondral phase I and collagen I phase in the bone phase. Anti-col I monoclonal antibodies (1:400) and anti col II (1:400) were used. The mouse and rabbit specific detection system HRP/DAB (ABC) detection IHC kit (ab64264) was used. The antibodies and the detection system were purchased from Abcam^®^, (Cambridge, MA, USA). The positivity was identified with 3, 3′ diaminobenzidine (DAB), and the nuclei were contrasted with Gill’s hematoxylin. Samples of articular cartilage and trabecular bone tissue were used as positive controls of the technique, and the primary antibody was omitted as a negative control. The samples were analyzed and also photographed using an Olympus AX70 microscope (Olympus, Tokyo, Japan).

### 4.12. Intraarticular Implantation Surgery

In order to reduce differences in the quality of tissue repair due to the genetic background of the animal, three different implants were placed on the same knee. Each lesion was 3 mm in diameter by 9 mm in length, reaching the subchondral bone; the defects were created in the center of the distal weight-bearing surface. The animal was anesthetized with intravenous xylazine (0.1 mg/kg) and Ketamine (7 mg/kg), and then placed in a lateral recumbent position. For surgery, a medial parapatellar approach to the joint capsule in its left hind leg, continuing to dislocate the patella laterally and expose the femoral condyles, was performed. A circular drill bit with a 6 mm diameter was used to perform the lesions to the subchondral bone. Three treatments were applied per knee as follow: Group 1: chondral monophasic scaffold, cellularized (AM); Group 2: biphasic scaffold, cellularized only in the chondral phase; and Group 3: biphasic scaffolding, cellularized in both the chondral and bone phases. In order to hold the graft onto the surrounding tissue, two drops of glue tisuacryl (Tisuacryl^®^, BIOMAT) were placed on the surface. Finally, two internal sutures were performed with polyglycolic acid (PGA Atramat^®^, International Pharmaceutical SA de CV Mexico, DF) in the synovial capsule, muscle and tendons, and with outer nylon (Vicryl 3-0 Ethicon, Inc.; Nylon ™ Suture ETHILON 3-0, Ethicon, Inc. Somerville, NJ) on the skin. During the immediate postoperative period, the animals were maintained in cages with standard care. After this period of time, the motion of the knee was not restricted and full weight-bearing on the surgical joints was allowed.

### 4.13. Ethics Statement

The protocol with the identification code P218-00337 involving research on animals was revised and approved by the UANL School of Medicine & University Hospital Institutional Review Board (October 2018), and experiments were conducted following the Mexican standard for the treatment of experimental animals (Norma Oficial Mexicana 062-ZOO-1999).

## 5. Conclusions

Our findings demonstrated the in vivo effectiveness of a biphasic and bioactive scaffold, based on silk fibroin, which intertwines thechondro-inductive features and biomechanical capability with an appropriate integration into the surrounding tissue; this represents a promising alternative for osteochondral tissue-engineering applications.

## Figures and Tables

**Table 1:** This is a table showing Young’s modulus via an unconfined compression assay.

Group	Description	Young’s Module (kPa)
A	Fibroin+NaCl	214.59
B	Fibroin/NaCl:BCM (1:1) 20–100 µm	805.01
C	Fibroin/NaCl:BCM (2:1) 20–100 µm	474.19
D	Fibroin/NaCl:BCM (3:1) 20–100 µm	554.14
E	Fibroin/NaCl:BCM (1:1) 10–20 µm	476.96
F	Fibroin/NaCl:BCM (2:1) 10–20 µm	301.59
G	Fibroin/NaCl:BCM (3:1) 10–20 µm	385.23

**Table 2:** Primers used for the qRT-PCR analysis.

Gene	Sequence 5′-3′
*SOX 9*	FW: AACGGCTCCAGCAAGAACAAG RV: GCTCCGCCTCCTCCACGAAG
*COL2A1*	FW: TCATCCAGGGCTCCAATGACGTG RV: AACAGTCTTGCCCCACTTACCG
*ACAN*	FW: CAACAATGCCCAAGACTACCAG RV: TTCCACTCGCCCTTCTCGTG
*COL10A1*	FW: CAGGAACTCCCAGCACGCAGA RV: CAGCGTAAAACACTCCATGAACCA
*MMP13*	FW: CGCCAGACAAATGTGACCCTT RV: AAAACAGCTCCGCATCAACC

## References

[1] O’Driscoll S. (1998). Current Concepts Review-The Healing and Regeneration of Articular Cartilage. J. Bone Jt. Surg..

[2] Ulrich-Vinther M., Maloney M.D. (2003). Articular cartilage biology. J. Am. Acad. Orthop. Surg..

[3] Mikos A.G., Herring S.W. (2006). Engineering complex tissues. Tissue Eng..

[4] Schinhan M., Gruber M. (2012). Critical-size defect induces unicompartmental osteoarthritis in a stable ovine knee. J. Orthop. Res..

[5] Shimomura K., Moriguchi Y. (2014). Osteochondral tissue engineering with biphasic scaffold: Current strategies and techniques. Tissue Eng. Part Rev..

[6] Liu M., Yu X. (2013). Tissue engineering stratified scaffolds for articular cartilage and subchondral bone defects repair. Orthopedics.

[7] Sah M., Pramanik K. (2010). Regenerated silk fibroin from B. mori silk cocoon for tissue engineering applications. Int. J. Environ. Sci. Technol..

[8] Wang Y., Kim H.J. (2006). Stem cell-based tissue engineering with silk biomaterials. Biomaterials.

[9] Li J.J., Kim K. (2015). Abiphasic scaffold based on silk and bioactive ceramic with stratified properties for osteochondral tissue regeneration. J. Mater Chem. B Mater Biol. Med..

[10] Yan L.P., Silva-Correia J. (2015). Bilayered silk/silk-nano CaP scaffolds for osteochondral tissue engineering: In vitro and in vivo assessment of biological performance. Acta Biomater..

[11] Voga M., Drnovsek N., Novak S., Majdic G. (2019). Silk fibroin induces chondrogenic differentiation of canine adipose–derived multipotent mesenchymal stromal cells/mesenchymal stem cells. J. Tissue Eng..

[12] Lin C.H. (2014). Evaluation of decellularized extracellular matrix of skeletal muscle for tissue engineering. Int. J. Artif. Organs.

[13] Ran F., Zhou M. (2015). Tissue-engineered grafts constructed using bone marrow derived mesenchymal stem cells and vascular acellular matrices. J. Biomater. Tissue Eng..

[14] Wang Q., Zhang C. (2014). The preparation and comparison of decellularized nerve scaffold of tissue engineering. J. Biomed. Mater. Res. A.

[15] Kun L., Yan Z. (2017). Development and characterization of oriented scaffolds derived from cartilage extracellular matrix. Hua Xi Kou Qiang Yi Xue Za Zhi.

[16] Moncada-Saucedo N.K., Marino-Martínez I.A.A. (2019). Bioactive Cartilage Graftof IGF1-Transduced Adipose Mesenchymal Stem Cells Embeddedin an Alginate/Bovine Cartilage Matrix Tridimensional Scaffold. Stem Cells Int..

[17] Gao Y., Liu S. (2014). The ECM-cell interaction of cartilage extracelular matrix on chondrocytes. Biomed. Res. Int..

[18] Almeida H. (2016). Fibrin hydrogels functionalized with cartilage extracellular matrix and incorporating freshly isolated stromal cells as an injectable for cartilage regeneration. Acta Biomater..

[19] Ge Z., Li C. (2012). Functional biomaterials for cartilage regeneration. J. Biomed. Mater Res. A.

[20] Haroun A.A., Gamal-Eldeen A. (2009). Preparation, characterization and in vitro biological study of biomimetic three-dimensional gelatin-montmorillonite/cellulose scaffold for tissue engineering. J. Mater. Sci. Mater. Med..

[21] Brigham M.D., Bick A. (2009). Mechanically robust and bioadhesive collagen and photocrosslinkable hyaluronic acid semi-interpenetrating networks. Tissue Eng. Part A..

[22] DeKosky B.J., Dormer N.H. (2010). Hierarchically designed agarose and poly (ethylene glycol) interpenetrating network hydrogels for cartilage tissue engineering. Tissue Eng. Part C Methods..

[23] Zhang Y., Zhang J., Chang F., Xu W., Ding J. (2018). Repair of full-thickness articular cartilage defect using stem cell-encapsulated thermogel. Mater. Sci. Eng. C.

[24] Nazarov R., Jin H. (2004). Porous 3-D Scaffolds from Regenerated Silk Fibroin. Biomacromolecules.

[25] Barlian A., Judawisastra H. (2018). Chondrogenic differentiation of adipose-derived mesenchymal stem cells induced by L-ascorbic acid and platelet rich plasmaon silk fibroin scaffold. Peerj.

[26] Kim U., Park J. (2005). Three-dimension alaqueous-derived biomaterial scaffolds from silk fibroin. Biomaterials.

[27] Brown C.F., Yan J., Han T.T., Marecak D.M., Amsden B.G., Flynn L.E. (2015). Effect of decellularized adipose tissue particle size and cell density on adipose-derived stem cell proliferation and adipogenic differentiation in composite methacrylated chondroitin sulphate hydrogels. Biomed. Mater..

[28] Tsai C.H., Chou M.Y. (2002). A composite graft material containing bone particles and collagen in osteoinduction in mouse. J. Biomed. Mater. Res..

[29] Chang C.H., Chen C.C. (2013). Human acellular cartilage matrix powders as a biological scaffold for cartilage tissue engineering with synovium-derived mesenchymal stem cells. J. Biomed. Mater. Res. Part A.

[30] Deng C., Zhu H., Li J., Feng C., Yao Q., Wang L. (2018). Bioactive scaffolds for regeneration of cartilage and subchondral bone interface. Theranostics.

[31] Teng B.H., Zhao Y.H., Wang L.Y., Yang Q., Li H.F., Li Y.J. (2018). Preparation and characterization of oriented scaffolds derived from cartilage extracellular matrix and silk fibroin. Hua Xi Kou Qiang Yi Xue Za Zhi.

[32] Rowland C.R., Colucci L.A. (2016). Fabrication of an atomically-shaped cartilage constructs using decellularized cartilage-derived matrix scaffolds. Biomaterials.

[33] Buckwalter J.A., Brown T.D. (2004). Joint injury, repair, and remodeling: roles in post-traumatic osteoarthritis. Clin. Orthop. Res..

[34] Muiznieks L.D., Keeley F.W. (2013). Molecular assembly and mechanical properties of the extracellular matrix: A fibrous protein perspective. Biochim. Acta.

[35] Han E.H., Chen S.S. (2011). Contribution of proteoglycan osmotic swelling pressure to the compressive properties of articular cartilage. Biophys. J..

[36] Chang C.H., Kuo T.F. (2006). Tissue engineering-based cartilage repair with allogen ouschondrocytes and gelatin-chondroitin-hyaluronan tri-copolymer scaffold: A porcine model assessed at 18, 24, and 36 weeks. Biomaterials.

[37] Pabbruwe M.B., Esfandiari E. (2009). Induction of cartilage integration by a chondrocyte/collagen-scaffold implant. Biomaterials.

[38] Kelly D.J., Crawford A. (2007). Biochemical markers of the mechanical quality of engineered hyaline cartilage. J. Mater. Sci. Mater. Med..

[39] Chen F.S., Frenkel S.R. (1999). Repair of articular cartilage defects: part I. Basic Science of cartilage healing. Am. J. Orthop..

[40] Yu H., Vandevord P.J. (2008). Promotion of osteogenesis in tissue-engineered bone by pre-seeding endothelial progenitor cells-derived endothelial cells. J. Orthop. Res..

[41] Matsumoto T., Kuroda R. (2008). Circulating endothelial/skeletal progenitor cells for bone regeneration and healing. Bone.

[42] Zavan B., Giorgi C. (2007). Osteogenic and chondrogenic differentiation: Comparison of human and rat bone marrow mesenchymal stem cells cultured into polymeric scaffolds. Eur. J. Histochem..

[43] Patra D., Sandell L.J. (2012). Antiangiogenic and anticancer molecules in cartilage. Expert Rev. Mol. Med..

[44] Huey D.J., Hu J.C. (2012). Unlike bone, cartilage regeneration remains elusive. Science.

